# Effects of Anthropogenic Disturbance and Seasonal Variation on Aerobiota in Highly Visited Show Caves in Slovenia

**DOI:** 10.3390/microorganisms11102381

**Published:** 2023-09-23

**Authors:** Rok Tomazin, Saša Simčič, Sanja Stopinšek, Andreja Nataša Kopitar, Andreja Kukec, Tadeja Matos, Janez Mulec

**Affiliations:** 1 Institute of Microbiology and Immunology, Faculty of Medicine, University of Ljubljana, Zaloška cesta 4, SI-1000 Ljubljana, Slovenia; rok.tomazin@mf.uni-lj.si (R.T.); sasa.simcic@mf.uni-lj.si (S.S.); andreja-natasa.kopitar@mf.uni-lj.si (A.N.K.); tadeja.matos@mf.uni-lj.si (T.M.); 2 Health Centre Hrastnik, Novi dom 11, SI-1430 Hrastnik, Slovenia; sanja.stopinsek@zd-hrastnik.si; 3 Department of Public Health, Faculty of Medicine, University of Ljubljana, Zaloška cesta 4, SI-1000 Ljubljana, Slovenia; andreja.kukec@mf.uni-lj.si; 4 Karst Research Institute, Research Centre of the Slovenian Academy of Sciences and Arts, Titov trg 2, SI-6230 Postojna, Slovenia; 5 UNESCO Chair on Karst Education, University of Nova Gorica, SI-5271 Vipava, Slovenia

**Keywords:** aerobiology, aerosols, show cave, anthropogenic effect, infection control

## Abstract

Aerosols in caves are natural tracers and, together with climatic parameters, provide a detailed insight into atmospheric conditions, responses to climatic changes and anthropogenic influences in caves. Microbiological air monitoring in show caves is becoming increasingly useful to understand changes in cave ecosystems and to implement and review measures for sustainable cave use and tourism development. In 2017 and 2018, air along tourist trails in caves Postojnska jama and Škocjanske jame (Slovenia) was sampled before and after tourist visits. Samples were analysed using culture-dependent methods, flow cytometry, detection of β-D-glucan and lipopolysaccharide and compared with CO_2_ and temperature data to measure anthropogenic influences and seasonality on aerobiota. While the presence of tourists significantly increased concentrations of airborne microorganisms (*p* < 0.05), β-D-glucan and CO_2_ did not show such a trend and were more dependent on seasonal changes. Locally, concentrations of cultivable microorganisms above 1000 CFU/m^3^ were detected, which could have negative effects on the autochthonous microbiota and possibly on human health. A mixture of bacteria typically associated with humans was found in the air and identified with MALDI-TOF MS. Using MALDI-TOF MS, we achieved a 69.6% success rate in identification. *Micrococcus luteus*, *Streptococcus mitis*, *Staphylococcus epidermidis* and *Moraxella* spp. were recognized as good indicators of cave anthropisation.

## 1. Introduction

Caves are specific, unique and fragile ecosystems due to their relative inaccessibility, constant microclimate, low availability of nutrients, limited capacity for self-purification, undiscovered biodiversity and the absence of some stressors, such as UV radiation and desiccation [1,2,3]. Karst caves are becoming increasingly interesting for tourists precisely because of their uniqueness. Human impact poses a major threat to caves and their conservation in some cases, through climatic changes (e.g., increased CO_2_ levels), inputs of organic nutrients and allochthonous microorganisms [1,2,4]. The presence of people in the cave can lead to irreversible changes in the composition of the biocoenosis [5,6]. Therefore, in the caves most frequented by tourists, such as Postojna Cave (Postojnska jama) and Škocjan Caves (Škocjanske jame) in western Slovenia, efforts are being made, such as disinfecting footwear before entering and limiting artificial lighting in the cave, to minimize the impact of anthropogenic changes and to operate these show caves sustainably [7,8,9,10,11]. Understanding the cave aerobiome is becoming increasingly important, especially because of its impact on biodeterioration of underground cultural heritage sites and the introduction of allochtonous, opportunistic and even pathogenic microorganisms [2,3,5,10,12]. Cave air, with all its properties, is an important natural resource that can be affected by tourists visiting show caves. On the other hand, the effects of bioaerosols on humans are still poorly understood [13,14]. The anthropisation of caves is studied with various microbiological methods. In addition to the standard cultivation of microorganisms, which is limited by its relatively low sensitivity and its dependence on media types and incubation conditions, other techniques are increasingly being used to complement culture-dependent techniques or as stand-alone methodologies for microbial biomass analysis [5,15]. These include metagenomic-based approaches, such as advanced molecular genetic techniques and high-throughput sequencing technologies, which greatly expand our picture of the structure and dynamics of microbial populations [16,17,18,19,20]. However, other culture-independent methods, such as flow cytometry, detection of cell wall and membrane structural components and ATP-based luminescence methods, have also proven to be very informative in cave microbiology [4,15,21,22,23]. In the study of show caves, flow cytometry alone or in combination with optical microscopy has been used for biofilm analyses, especially for phototrophic biofilms and also for aerobiological studies [21,24,25,26]. To the best of the authors’ knowledge, the detection of cell wall and membrane structure components of bacteria and fungi in caves has so far only been carried out in the Postojnska jama [4]. These components, mainly lipopolysaccharide (LPS) and β-D-glucan (BDG), are known triggers of innate immunity and have immunomodulatory effects [27,28]. LPS is an important component of the outer membrane of Gram-negative bacteria and is responsible for the toxic properties (called endotoxin) of many bacterial species [27,29,30]. BDG, on the other hand, is the main structural component of the fungal cell wall. It is less immunogenic than LPS and is used as a panfungal biomarker in the diagnosis and prognosis of invasive disseminated mycoses [31,32]. Their detection in clinical samples is included in current diagnostic guidelines, as they play an important role in the pathobiology of many infectious diseases and intoxications [33]. Since LPS and BDG have also been detected in cave air [4], these two molecules could be used as indicators of aerosolised bacteria and fungi. The aim of this study was to assess the anthropogenic effect of mass tourism on the air microbiota and consider its potential impact on human health in Postojnska jama and Škocjanske jame. In addition to the standard culture-based approach and flow cytometry, we also included the detection of subcellular components BDG and LPS to determine their potential use as biomass markers in show caves.

## 2. Materials and Methods

### 2.1. Sampling Sites

**Figure 1:**
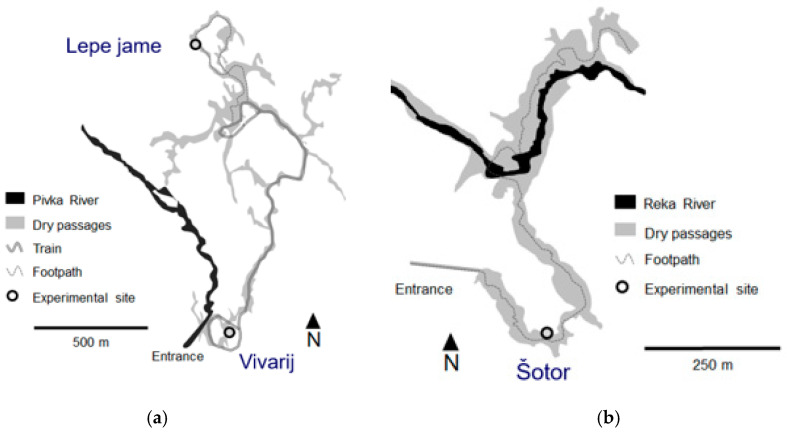
Sampling sites in Postojnska jama (**a**) and Škocjanske jame (**b**). The ground plan is adapted from the Cave cadastre of the Karst Research Institute, Research Centre of the Slovenian Academy of Sciences and Arts and Speleological Association of Slovenia.

The air was sampled in two show caves in Slovenia. The Postojnski jamski sistem (Postojna Cave System, 45°46′57″ N, 14°12′13″ E, 529 m a.s.l.), formed in Cretaceous limestone [34], consists of climatically distinct parts, some of which are subject to hourly variations [35]. The greatest climatic fluctuations occur along the underground Pivka River and at the entrances. The entire cave system is 24 km long and includes five historical caves with separate entrances (Postojnska jama, Otoška jama, Magdalena jama, Črna jama and Pivka jama). The size of the underground passages, the cave formations, the historical significance, a regular underground tourist train and its reputation as a cradle of speleobiology research attracted more than 800,000 tourists annually (2018) before the SARS-CoV-2 pandemic in 2020–2021. After the pandemic, the number of tourists is increasing again, approaching pre-pandemic figures with more than 600,000 visitors in 2022. The 5.3 km tour of the cave is made up of a 3.5 km round trip on a tourist train and a 1.8 km walk. The electric train runs inside the cave and picks up tourists near the entrance and takes them to the start of the guided tour. At the end of the tour, the train returns to the exit where the tour ends. Each train has a maximum of 16 carriages with a maximum of 116 passengers on board and reaches a maximum speed of 10 km per hour. In a separate section of Postojnska jama, tourists (150,000 in 2018) visit the Vivarium with a speleobiological exhibition featuring the most typical cave animals. Two sampling sites were selected in Postojnska jama for this study along the tourist trails, one of which was in the Vivarium, about two metres from the tourist trail. In this part of the cave, tourists stay in smaller groups for different periods of time to observe the collection of different live cave animals. The other location was in a section called Lepe jame (literally Beautiful Caves) at the lowest point of the tourist visit, three metres from the footpath. Tourists come in large groups and stay for a short period of a few minutes to listen to the explanations of the cave guides (Figure 1a).

The Škocjanske jame (Škocjan Caves, 45°39′53.33″ N, 13°59′40.44″ E) were formed by the Reka River in Cretaceous and Paleocene limestones [36,37] and are 6.2 km long with large galleries, chambers and an underground canyon. The Škocjanske jame are an underground karst wetland according to the Ramsar Wetland Classification System, part of Natura 2000 and listed on the UNESCO World Heritage List. Like Postojnska jama, Škocjanske jame are open to tourists all year round (except during the SARS Cov-2 epidemic in 2020–2021) and attract many visitors (180,000 in 2018) on a 2.6 km footpath. The aerosol sampling site was located in the section called Šotor (literally Tent), about three metres from the tourist footpath in the Tiha jama section. Tourists pass by this site in large groups and occasionally stay for several minutes to listen to the cave guides’ explanations (Figure 1b). The distance of three metres between the sampling station and the tourists’ footpath in both caves was chosen to avoid direct contact of the tourists with the sampling station and the sampling procedure and to allow a normal passage of the tourist groups.

### 2.2. Air Sampling and Measurement of Environmental Parameters

Air sampling began in the morning, a few hours before the caves were opened to tourists, to obtain background samples and atmospheric conditions. Immediately after the arrival of the tourist groups, the second round of sampling started to observe a direct impact of the tourists on the cave climate and to record the number of tourists. Measurements and aerosols were collected 1.5 m above the ground on a portable platform. Air sampling and measurements in Postojnska jama in the Lepe jame section were carried out: 31 January 2017, 23 March 2017, 25 May 2017, 22 August 2017, 3 October 2017, 5 December 2017, 28 August 2018, 28 November 2018 and in the Vivarium: 23 March 2017, 25 May 2017, 22 August 2017, 3 October 2017, 5 December 2017, 29 August 2018, 28 November 2018. Measurements and air samples were collected in Škocjanske jame: 1 February 2017, 22 March 2017, 24 May 2017, 23 August 2017, 4 October 2017, 6 December 2017, 30 August 2018, 29 November 2018. A Coriolis^®^µ Cyclonic Air Sampler (Bertin Technologies, Saint Quentin en Yvelines, France) with an airflow of 150 L/min was used to collect aerosols. Airborne particles were collected in 10 mL of sterile saline (0.9% NaCl) from a total air volume of 4.5 m^3^. Prior to sampling, a flow tube was disinfected with 96% ethanol. After sampling in the cave, the fluid was aseptically divided in a laboratory to perform individual microbiological analyses, culture-independent and culture-dependent biomass estimates and microbial identifications. Sample losses due to evaporation during sampling were not considered, as the cave atmosphere reached 100% air saturation.

In parallel with sampling, temperature and relative humidity were measured continuously with a Kestrel 4500 PocketWeather portable tracker (Boothwyn, PA, USA), and atmospheric CO_2_ was measured with an MI70 Vaisala handheld carbon dioxide metre (Vaisala, Vantaa, Finland) with a data acquisition time of 30 s for both instruments.

### 2.3. Biomass Estimators and Microbial Identification

#### 2.3.1. Total Cell Counts and Cell Viability

Total cell counts and cell viability were determined using the Cell Viability Kit with Liquid Counting Beads (BD Biosciences, Franklin Lakes, NJ, USA) according to the manufacturer’s instructions on a FACS Canto Flow Cytometer (Becton Dickinson, Franklin Lakes, NJ, USA). Data analysis was performed using the software BD FACSDiva 6.1.2 (Becton Dickinson). The results were expressed as microbes per cubic metre.

#### 2.3.2. Detection of (1→3)-β-D-Glucan (BG)

The Fungitell assay (Fungitell, Associates of Cape Cod, Falmouth, MA, USA) was used to determine BDG concentration. A portion of the liquid samples was analysed in duplicate, and results were expressed as mean values (pg/mL) and calculated per volume of air (pg/m^3^). If a categorical discrepancy was found between replicate samples with a standard deviation of 20 pg/mL or more, the samples were repeated. The tests were performed according to the manufacturer’s instructions, except for the interpretation of the results, as the assay is only validated for blood samples.

#### 2.3.3. Detection of Lipopolysaccharide (LPS)

The Limulus Amoebocyte Lysate Assay (LAL, Associates of Cape Cod, Falmouth, MA, USA) was used to quantify LPS, the endotoxins of Gram-negative bacteria. Control standard endotoxin was used to prepare dilutions of standard endotoxin from which the standard curve was constructed. Results were expressed as mean values in endotoxin units per millilitre (EU/mL) and calculated per volume of air (EU/m^3^). The tests were performed according to the manufacturer’s instructions, except for the interpretation of the results, as the assay was only validated for blood samples.

#### 2.3.4. Microbial Cultivation

A 1.5% nutrient agar (NA, Sigma-Aldrich, St. Louis, MO, USA) was used to estimate and identify the cultivable fraction of airborne microorganisms, as is common in microbiology and has been used in previous studies in Slovenian caves [3,4] (Mulec, 2014; Mulec et al., 2017). A portion of the liquid samples was evenly distributed on the NA plates, and then, the plates were incubated at 37 °C for 48 h and at 20 °C for 7 days. In addition to the NA plates, 5.0% defibrinated sheep blood agar (BA) and thioglycolate broth (THI) (Becton Dickinson, USA) were also used as primary isolation media. The inoculated BA and THI were incubated at 37 °C for 7 days. Colony forming units (CFU) counted were expressed per volume of air (CFU/m^3^) according to the equation provided by the manufacturer (Bertin Technologies, Saint-Quentin-en-Yvelines, France). Distinct morphotypes from all primary selection agar media were inoculated onto blood agar (BA) and incubated for 24 to 48 h at 37 °C for subsequent identification with MALDI-TOF MS (Matrix-Assisted Laser Desorption/Ionisation Time-Of-Flight Mass Spectrometry).

#### 2.3.5. Microbial Identification

The pure microbial isolates on the BA plates were identified using MALDI-TOF MS with a formic acid on-spot extraction technique. A colony 24 to 48 h old was spread on the MALDI steel plate and overlaid with 1 µL of 98% formic acid. After drying, the sample was covered with 1 µL of photoabsorbent saturated α-cyano-4-hydroxycinnamic acid (HCCA) matrix solution in 50% acetonitrile-2.5% trifluoroacetic acid (Bruker Daltonik, Bremen, Germany) and allowed to dry before subsequent analysis using a Linear-Mode microflex LT/SH MALDI-TOF MS system (Bruker Daltonik). The spectra obtained were analysed using the MALDI-TOF Biotyper^®^ (MBT) Compass software version 4.1 (Bruker Daltonik). The Bruker bacterial test standard was used for calibration according to the manufacturer’s instructions. The quality of identification was evaluated using the score from 0 to 3 given by the manufacturer. A score ≥2.00 indicated reliable identification at species level, a score of 1.70 to 1.99 indicated reliable identification at genus level and a score of <1.70 was interpreted as no identification.

### 2.4. Statistical Analysis

Descriptive statistics were used to present the distribution of absolute values of environmental parameters (temperature and CO_2_ concentration), quantification of airborne microorganisms and biomass estimators in the observed caves before and after the tourists’ visit. Average values are given for temperature and CO_2_ concentration. The influence of tourists on the measured variables (concentration of microorganisms, CO_2_ and BDG) was determined using the Wilcoxon Signed Rank Test adjusted by the Bonferroni correction; *p*-values of less than 0.05 were considered statistically significant. Statistical analyses were performed using IBM^®^ SPSS^®^ for Windows version 26 (SPSS Inc., IBM Company, Armonk, NY, USA) and Excel 2016^®^ for Windows^®^ (Microsoft™).

## 3. Results

### 3.1. Environmental Parameters, Quantification of Airborne Microorganisms and Biomass Estimators

Table 1 shows the results for air temperature, CO_2_ and BDG concentrations and microorganisms present in the air obtained by cultivation and flow cytometry in the observed caves before and after the tourists’ visit. The highest concentration of cultivable microorganisms, regardless of the type of culture medium and incubation conditions, was considered as the final result in Table 1, Table 2 and Tables S1–S5. The LPS concentrations were not reported due to the low sensitivity of the test: 82.6% of the measurements were below the detection limit (<0.005 EU/mL), especially for the “before tourists” samples compared to the “after tourists” samples, namely 95.7% (22/23) and 69.6% (16/23), respectively, and were not analysed further.

Measured CO_2_ concentrations ranged from 520–2390 ppm before tourist visits and from 510–2110 ppm after visits, with concentrations greater than 1000 ppm measured at all sampling sites from May to October (Table 1). The highest “after tourist” concentrations were observed at sites that already had high “before tourists” concentrations. CO_2_ concentrations generally increased in both caves after the tourists’ visits, but not in a statistically significant way (Table 2). Relative humidity reached 100% air saturation during all sampling campaigns.

Using flow cytometry, the highest concentrations were recorded in November 2018 in Šotor and Lepe jame and in March 2017 in the Vivarium with 105,733 microbes/m^3^, 74,396 microbes/m^3^ and 69,722 microbes/m^3^, respectively (Table 1). Microbial viability ranged from 30.2–95.5% in the “before tourists” samples and from 57.6–96.5% in the “after tourists” samples, with an average viability of 79.9% (Table 1). Higher concentrations were found mainly (73.9% (17/23)) in the “after tourist” samples. The highest “after tourist” concentrations were observed at sites that already had high “before tourist” concentrations (Table 1). This indicates a continuous microbial input from tourists and that the microbes remained aerosolized and they did not settle between tourist visits, especially in Postojnska jama during the summer months in the peak tourist season in summer. Here, the changes in concentrations before and after tourist visits were not statistically significant (Table 2).

The highest concentrations of cultivable microorganisms In Postojnska jama were measured in the Vivarium in May 2017 and November 2018 and in Lepe jame in August 2017 with 1287 CFU/m^3^, 920 CFU/m^3^ and 787 CFU/m^3^, respectively (Table 1). In Šotor (Škocjanske jame), the highest concentration was measured in October 2017 at 539 CFU/m^3^ (Table 1). Similar to flow cytometry, the highest concentrations were usually found in the samples “after the tourists”. This was the case in 78.3% (18/23) of the cases while higher concentrations were found in 21.7% (5/23) in the “before tourists” samples. The concentrations of the “after tourists” samples were 1.8 to 10.2 times higher than those of the “before tourists” samples in Lepe jame, 1.2 to 8.5 times higher in the Vivarium and 1.5 to 6.6 times higher in Šotor (Tables S1–S4). BDG concentrations in the “after tourists” samples were higher in only half of the samplings (52.2%, 12/23), with concentrations between 1.1 and 6.0 times compared to those of the “before tourists” samples (Table 1).

The Influence of tourists on the measured variables (median concentration of microorganisms, CO_2_ and BDG) is shown in Table 2. As we can see, tourists have a statistically significant influence on the median concentration of cultivable microorganisms only after tourists visit Šotor (*p* = 0.046). In the other cases, the influence of the cave visitors was not statistically significant (*p* > 0.05).

### 3.2. Identification of Airborne Microorganisms

From January 2017 to November 2018, 11,711 bacterial and fungal isolates were obtained from all three sampling sites and are presented in Tables S1–S5. A total of 51 bacterial species from 28 genera and 9 fungal species from 7 genera were identified (Tables S1–S3). A large number of isolates, 55.8% (2174/3896) from the “before tourists” samples and 33.1% (2583/7815) from the “after tourists” samples, could not be identified due to low MALDI scores (<1.70) (Table 3). More isolates were identified in the “after tourists” samples than in the “before tourists” samples: 60.0–69.6% compared to 42.0–49.2% (Table 3).

The most common genera of *Firmicutes* were *Bacillus* and *Staphylococcus*, accounting for up to 3.7% of the detected microorganisms in “before tourists” samples and up to 9.0% in the “after tourists” samples (Table S4). Among the *Actinobacteria*, *Micrococcus* and *Arthrobacter* dominated with 17.4% and 3.2% of the “before tourists” isolates and 29.3% and 2.6% of the “after tourists” isolates, respectively. Micrococci were indeed the most frequently isolated bacteria from all sampling sites (Table S4). The most common genera of *Proteobacteria* were *Pseudomonas* and *Acinetobacter*, which accounted for 2.9% and 0.6% of the isolates in the “before tourists” samples and 1.5% (*Pseudomonas* spp.) in the “after tourists” samples (Table S4). We identified five different *Pseudomonas* spp. in concentrations ranging from 17–33 CFU/m^3^ and three species of *Acinetobacter* spp. in a range of 7–99 CFU/m^3^ (Table S4). In Lepe jame and Šotor, *M. luteus* was the bacterial species that reached the highest concentrations before the tourist visits, 134 CFU/m^3^ and 67 CFU/m^3^, respectively, while in the Vivarium the highest concentration before the tourist visits was reached by *Aspergillus versicolor*, 217 CFU/m^3^ (Table S2). After the tourist visit, the highest concentrations at all three sampling sites were due to *M. luteus*, with 251 CFU/m^3^ in Lepe jame, 618 CFU/m^3^ in the Vivarium and 92 CFU/m^3^ in Šotor (Tables S2–S4). In addition to *A. versicolor*, eight other fungal species were detected in both caves at concentrations ranging from 2–217 CFU/m^3^ before the tourist visits and from 1–267 CFU/m^3^ after the visits (Table S5). The Šotor sampling site in the Škocjanske jame was the only sampling site where we could not detect any cultivable fungi before the tourist visits while in the Postojnska jama, fungi accounted for at least 4.2% of all detected microorganisms in the “before tourist” samples (Table S4). After the tourist visits, fungi were present at all sampling sites, accounting for 0.9–13.9% of the microorganisms detected (Table S5). Five species belonged to the phylum *Ascomycota* and four to the phylum *Basidiomycota*. Besides *A. versicolor*, two other species of filamentous fungi have been isolated: *A. fumigatus* and *P. variotii*, both of which were found only in the Lepe jame sampling site. Among the yeasts, the genera *Cutaneotrichosporon*, *Naganishia* and *Rhodotorula* were isolated from both caves while *Debaryomyces* was isolated only from Postojnska jama (Table S5).

### 3.3. Microbial Indicators of Cave Anthropisation

Among the 60 species of bacteria and fungi identified (Tables S4 and S5), we identified up to 14 species associated with the human microbiota that could serve as indicators of cave anthropisation (Table 4). Half of these microorganisms (7/14) belong to the genus *Staphylococcus*. Others are also part of the normal skin or oral microbiota, as these body parts are in direct contact with the external environment and can easily excrete into it. *S. epidermidis* and *S. warneri* as well as *M. luteus*, unlike others, were isolated from all three sampling sites and are considered strong anthropogenic indicators. Isolates of interest were also the viridans streptococci of the *S. mitis* species complex isolated from both sampling sites in Postojnska jama (Tables S1 and S2). They were identified as *S. mitis* and *S. pseudopneumoniae*. According to the American Biological Safety Association, all the bacteria mentioned belong to risk groups 1 or 2 because they have a limited pathogenic potential: 6/14 isolates belong to risk group 2 and 1/14 to risk group 1 while others (7/14) cause infections so rarely that they have not been assigned to any risk group (Table 4). Among the fungi, the basidiomycetous yeasts *N. diffluens*, *N. liquefaciens* and *R. mucilaginosa* could indicate anthropisation of the cave.

## 4. Discussion

### 4.1. Microclimatic Parameters and β-(1,3)-D-Glucan

Large groups of people in karst caves affect microclimatic parameters. The influence of tourists on the maximum CO_2_ concentration and cave air temperature in Postojnska jama has already been studied [11,38,39]. The results show a clear correlation between tourist visits and the increase in CO_2_ and temperature when measured continuously while sporadic measurements did not show this correlation, which is explained by the natural ventilation of the caves [11,38,39]. Our results are consistent with this, as the field measurements show that CO_2_ generally increase slightly during tourist visits, 1.2 times the concentration before tourist visits, similar to the 1974 study by Gams [38]. However, in agreement with other studies [39,40], CO_2_ concentrations show a seasonal distribution, being highest in summer and autumn and lowest in winter, and were not as clearly affected by tourists as the aerobiome. BDG also showed a seasonal distribution with the highest concentrations in autumn and the lowest in spring at all sampling sites. One of the explanations for this is that concentrations of airborne fungi, especially necrotrophic plant pathogens, such as *Aspergillus* spp., peak in the autumn months [41,42]. Moreover, since both caves studied draw in outside air during the cooler season (chimney effect) [11], BDG concentrations may be higher than in other seasons. A good example is the Vivarium, which had high concentrations of *A. versicolor* (a possible source of BDG) in the samples in November 2018. However, in order to assess the seasonal effect in the observed caves, a time-series analysis of the observed parameters CO_2_ and BGD has to be performed. Nevertheless, based on our results, we conclude that BGD is not a reliable indicator of cave anthropisation. Due to the limitations of our study in terms of sample size, further research is needed to confirm this.

### 4.2. Airborne Microorganisms and Tourist Visits

As already shown by other studies [3,5], higher concentrations of aerosolised microorganisms were generally detected after tourist visits by both flow cytometry and culturing. However, this was not the case for about a quarter of the samples, probably because cave visits are very frequent in Škocjanske jame and especially Postojnska jama and the disturbed and introduced microorganisms do not have time to settle between visits. Higher microbial concentrations were associated with tourist visitation but not with the number of tourists, e.g., when the highest numbers of cutivable microorganisms were observed in the Vivarium in May 2017 and November 2018, only 2–25 tourists passed by the sampling site. On the other hand, when a group of 358 tourists visited Lepe jame in October 2017, only 68 CFU/m^3^ were detected. In general, tourist visits increased microbial concentrations by 1.2 to 10.2 times the concentration measured before the tourist visit. A similar trend was observed in Škocjanske jame and also in four Romanian show caves (Ursilor, Muierilor, Meziad and Polovragi), where the detected microbial concentrations were also similar [3,5].

As Postojnska jama and Škocjanske jame are highly visited tourist attractions in western Slovenia, it is difficult to determine the natural state of aerobiota that is not influenced by tourists. The COVID-19 pandemic period, when the caves were closed to visitors, would have been a perfect control, but unfortunately, no sampling was carried out due to pandemic-related restrictions.

### 4.3. Human-Associated Microbiota and Ecological Disturbance

In 2018, Barcea et al. proposed five ecological disturbance categories based on Porca et al.’s study on early detection and control of fungal outbreaks in show caves [5,43]. These categories are based on measured bacterial and fungal concentrations in cave air and their potential impact on the cave ecosystem [5,43]. The highest category, Category 5, when concentrations are >1000 CFU/m^3^, is associated with irreversible ecological disturbance while the lowest category, Category 1, when concentrations are <50 CFU/m^3^, is associated with no anthropogenic impact on cave ecology at all [5,43]. In our case, a concentration of >1000 CFU/m^3^ was detected in the Vivarium in May 2017 after 25 tourists passed the sampling site. According to other studies, such high microbial concentrations can be associated with irreversible ecological disturbances caused by the presence of humans [5,43,44,45]. Other sampling sites did not show such high microbial loads and, therefore, cannot be associated with ecological damage, but they indicate a high input of allochthonous microorganisms into the cave ecosystem [5,43]. As the Postojnska jama and Škocjanske jame are large and well-ventilated, we assume that these high concentrations are only temporary and can be reduced or eliminated relatively quickly so that the impact on the cave ecology is probably limited, similar to the Nerja Cave in Spain or the Kinderlinskaya Cave in Russia [46,47,48].

In our study, we obtained data on the air microbiota in the caves every two months. This limits the insight into the structure and dynamics of the air microbiota. Therefore, in order to assess ecological disturbances more accurately, microbiological time series analyses need to be conducted.

### 4.4. MALDI-TOF MS Identification Success Rate

The authors agree that MALDI-TOF MS provides accurate identification results but sometimes with a lower success rate than in clinical microbiology; when analysed in a clinical setting, the success rate is >90% for different groups of microorganisms, but when analysed in caves, it rarely reaches 90% [3,49,50]. In our study, similar to others [3,50], we were unable to identify a certain proportion of isolates using the MALDI-TOF MS method, indicating their environmental origin. Interestingly, the proportion of unidentified isolates in the “before tourists” samples was different from the “after tourists” samples; we were able to identify 44.2% of isolates in the first group and 66.9% in the second. One of the explanations for this is that human-associated microorganisms, such as *Staphylococcus* spp. and *Micrococcus* spp., were present in greater proportions in the “after tourist” samples. Similar results were obtained in our previous study in Škocjanske jame, where only about 50% of the isolates were identified also [3]. A lower success rate in identifying cave isolates suggests that commercially available databases cover only a limited number of species that are not clinically relevant or are rarely associated with humans. Our results show that identification based on MALDI TOF-MS is sufficient when studying microorganisms associated with humans, but when the full spectrum of microorganisms is to be identified, PCR-based methods are recommended.

### 4.5. Microorganisms and Potential Indicators of Human Impact

As in other studies, bacteria from the phyla *Actinobacteria*, *Firmicutes* and *Proteobacteria* were frequently isolated from the samples [3,13,16,51,52]. We identified 51 different bacterial species, which is consistent with other culture-based studies, some of which found slightly lower species diversity in both sediments and air [44,53]. In the study by Dominguez-Moñino et al., 1.8–2.5 times more bacterial species were detected in the air than in our study [44]. These large differences are most likely due to the identification methods used, namely that Dominguez-Moñino et al. identified their isolates by sequencing the 16S rRNA gene, whereas we used MALDI-TOF MS, which resulted in significantly fewer species being identified. If we lower the Bruker cut-off value for a reliable species identification from 2.00 to 1.70, we get 84 different species, which is comparable to the show caves Gruta de las Meravillas and Cueva del Tesoro in southern Spain with 94 and 130 species, respectively [44]. Therefore, MALDI-TOF MS can be considered a suitable method for a rapid and cost-effective assessment of species diversity. In caves, greater species diversity is associated with the anthropisation of the cave, as is the presence of some bacterial species [4,44,54].

Many different species of bacteria and fungi have already been proposed as anthropogenic indicators. Some species could be specific to each cave, indicating the complexity and uniqueness of cave ecosystems, but researchers in all caves mentioned certain species that represent a common anthropogenic point. These are mainly coagulase-negative staphylococci, certain lactic acid bacteria and fungi associated with the skin microbiota [3,4,16,44,54,55]. In Postojnska jama and Škocjanske jame, some of the isolated microorganisms, e.g., *Debaryomyces*, *Rhodotorula* and *Dermacoccus*, are associated with the normal human microbiota while we rarely associate others with it [56]. These genera could potentially be indicators of human presence, but unfortunately, they lack the desired frequency of isolation or human exclusivity. An ideal indicator microorganism should be associated with humans, be easily detectable and always occur in relatively high concentrations or proportions when humans are/were present [54]. These criteria are best met by *M. luteus*—a member of the normal skin microbiota that has simple growth requirements and is isolated in high proportions at all sampling sites and at all times of the year, similar to other aerobiological studies in show caves [3,44,57,58]. *M. luteus* was also the most frequently isolated bacterium in the “before tourist” samples; we suspect that this is due to mass tourism in the two show caves in question, especially Postojnska jama. Both caves are main tourist hotspots in Slovenia, visited by hundreds of tourists daily (150,000–800,000 visitors/year), and it is possible that microorganisms associated with humans do not settle or are removed between visits. The second indicator candidate would be the *Streptococcus mitis* species complex detected in Postojnska jama. The *S. mitis* species complex is found exclusively in the oral cavity, making it a good anthropogenic indicator [56,59,60]. The *S. mitis* species complex has also been found in other show caves, indicating the anthropisation of the cave [16]. The third indicator should be *Staphylococcus epidermidis*, a member of the human skin microbiota [61], which was also detected at all sampling sites and accounted for up to 9.0% of all microorganisms detected. In addition to *S. epidermidis*, seven other coagulase-negative *Staphylococcus* species were isolated from the air after the tourist visits, three of which, *S. haemolyticus*, *S. lugdunensis* and *S. saprophyticus,* were isolated exclusively from the “after tourists” samples and could serve as anthropogenic indicators, as already suggested in some other studies [4,54]. Some other microorganisms that were not isolated in large quantities but are exclusively associated with humans could also indicate the presence of humans. A good example is *Moraxella* spp.—Gram negative diplococci normally found on mucosal surfaces and isolated in small quantities only in Lepe jame after tourist visits. Among the *Fungi*, the genera *Cutaneotrichosporon*, *Naganishia* and *Rhodotorula* were isolated from both caves while *Debaryomyces* was isolated only from Postojnska jama. These species are associated with the human microbiota, mainly as colonisers of the skin and intestinal mucosa [62,63,64]. These yeasts have been detected in the sediments and air of other European and South American caves, often in association with bat guano, so their role as anthropogenic indicators is not as clear as that of some bacteria [12,13,65,66,67,68].

In our study, we only monitored aerobiota, but to better understand the state of anthropisation, we would also need information on microorganisms on surfaces—both those in close contact with tourists and those far from tourist footpaths. This would give us the most realistic insight into the cave microbiota and its changes at the expense of tourism.

### 4.6. Opportunistic Pathogens in Cave Aerobiota

Among the previously discussed airborne microorganisms, some are considered both commensals and opportunistic pathogens [56], but no primary pathogens were found. All isolated bacteria and fungi belong to risk groups 1 or 2 because they have only limited pathogenic potential, or they do not belong to any risk group at all because they do not usually cause infections in the immunocompetent people. *Aspergillus fumigatus*, *Aspergillus versicolor* and *Paecilomyces variotii* isolated from the two caves studied can rarely cause forms of hypersensitivity reactions—allergic rhinitis, conjunctivitis and allergic bronchopulmonary aspergillosis [69,70]. These allergic reactions occur mainly in people with atopy or chronic respiratory diseases, such as asthma, cystic fibrosis and chronic obstructive pulmonary disease. Inhalation of conidia may trigger an acute exacerbation in these patients [69,70]. However, as mentioned above, Postojnska jama and Škocjanske jame are large and well-ventilated, which helps to redistribute and dilute airborne microorganisms relatively quickly, limiting exposure to moulds and their antigens to a short period of time.

There is no generally accepted limit for total microbial concentration to interpret the effects on human health; the results can only be compared with various values recommended in the literature. The WHO recommends that total indoor microbial concentrations should not exceed 1000 CFU/m^3^ [71], Rao et al. recommend ≤750 CFU/m^3^ [72], and the European Commission (EC) recommends ≤500 CFU/m^3^ for non-industrial premises [73]. If we apply the cut-off value recommended by WHO, only the situation in the Vivarium in May 2017 poses a risk of a potentially increased health risk, as concentrations of more than 1200 CFU/m^3^ were measured here. However, if the cut-off of Rao et al. is applied, the total concentrations in the Vivarium exceed the recommended value in 3/8 samplings and in 1/8 samplings in Lepe Jame. These high microbial loads in the Vivarium are probably the result of a smaller, less ventilated chamber compared to Lepe jame and Šotor, where microbial loads never exceed 1000 CFU/m^3^. Moreover, the way the Vivarium is visited differs from the other parts of Postojnska jama and Škocjanske jame: in the Vivarium, visitors look at the cave animals presented in terrariums and, therefore, stay longer in the Vivarium while in Lepe jame and Šotor, they are much more active, walk through the cave chambers and do not stay long at individual points despite the explanations of the guides. The Vivarium was also the only sampling site to exceed the recommended cut-offs prior to tourist visits, further confirming the Vivarium’s unique situation.

## 5. Conclusions

The results show that mass tourism in caves significantly changes the composition of the aerosol: higher total concentrations of microorganisms with a high number of species typical of the human microbiota. We have shown that MALDI-TOF MS is a reliable tool for identifying microorganisms associated with humans and is therefore suitable for assessing the anthropisation of caves. Postojnska jama and Škocjanske jame are large and well-ventilated, so we assume that these high concentrations are temporary and can be reduced or eliminated relatively quickly so that the impact on cave ecology and the likelihood of a visitor’s health being adversely affected during a visit is likely to be minimal or negligible. This was the first time β-D-glucan had been used in cave air samples to monitor anthropogenic and seasonal impacts on cave aerobiota. It turned out to be a poor anthropogenic marker but a better seasonal marker. To determine a better insight into the structure and dynamics of the air microbiota due to mass tourism, continuous microbiological monitoring should be carried out over a long period of time.

## Figures and Tables

**Table 1:** Temperature, CO_2_ and β-(1,3)-D-glucan concentrations and total microbial concentrations in air samples from January 2017 to November 2018 in Lepe jame (Postojnska jama), Vivarium (Postojnska jama) and Šotor (Škocjanske jame) before and after tourists’ visit.

		Before Tourist Visitation						During/After Tourist Visitation						
Location	Date	T	CO_2_	Microbes (Culture)	BDG	Total Microbes (Flow Cytometry)	% Live Microbes (Flow Cytometry)	T	CO_2_	Microbes (Culture)	BDG	Total Microbes (Flow Cytometry)	% Live Microbes (Flow Cytometry)	No. of Tourists
		(°C)	(ppm)	(CFU/m^3^)	(pg/m^3^)	(Microbes/m^3^)		(°C)	(ppm)	(CFU/m^3^)	(pg/m^3^)	(Microbes/m^3^)		
**Lepe jame**	31 January2017	10.7	560	51	<7.8	6556	88.1	10.6	540	188	<7.8	6622	86.2	91
	21 March 2017	10.7	660	93	<7.8	30,600	85.2	10.6	690	169	<7.8	39,289	84.1	136
	25 May 2017	10.8	1190	67	10	10,356	90.8	10.7	1230	51	19.1	12,800	88.5	135
	22 August 2017	10.8	1410	400	26.5	41,863	88.4	10.9	1550	787	30.6	41,919	89.3	154
	3 October 2017	10.9	2040	113	90.9	22,768	85.2	11	2000	68	151.2	8145	77.9	358
	5 December 2017	10.7	790	421	409.3	9111	81.9	12.3	800	327	584.7	6346	70.1	144
	28 August 2018	10.8	1580	17	85.2	10,616	76.1	11	1330	173	513.9	7976	71.3	602
	28 November 2018	10.4	1630	40	114.9	8321	70.9	10.5	1430	100	252.5	74,396	80.4	109
**Vivarium**	21 March 2017	12.5	660	143	<7.8	49,622	83.2	-	-	326	<7.8	69,822	63.5	0
	25 May 2017	12.7	1150	152	16.2	8888	86	10.7	1240	1287	18.5	13,426	80.7	25
	22 August 2017	13.9	1410	74	32.7	41,756	87.9	10.9	1650	333	26.8	46,451	83.7	246
	3 October 2017	13.1	760	643	<7.8	5348	74.2	11	850	786	30.1	9145	79.4	63
	5 December 2017	13.6	600	322	191.9	28,567	80.8	12.3	640	59	348.3	14,868	79.3	0
	28 August 2018	12.7	1350	11	62.5	6183	78.1	12.1	1410	81	157.9	6926	67.3	18
	28 November 2018	12.2	640	229	141.6	9157	81.5	11.9	750	920	<7.8	15,088	75.8	2
**Šotor**	1 February 2017	12.5	540	215	40.8	21,378	95.5	12.3	540	327	<7.8	24,311	96.5	7
	22 March 2017	12.6	810	65	8.4	25,911	84.4	12.4	820	60	<7.8	23,667	86.6	97
	24 May 2017	12.7	1300	51	8.9	10,454	90.4	12.5	1330	335	<7.8	10,319	84.9	119
	23 August 2017	12.6	1910	251	59	45,272	88.2	12.7	1950	459	17	44,728	86.2	182
	4 October 2017	12.7	1880	393	1326	14,800	78	12.5	1920	539	1437.7	6873	70.6	79
	6 December .2017	12.4	520	20	592.2	4882	77.1	12.4	510	122	296.5	7170	71.5	12
	29 August 2018	12.9	2390	77	185.4	25,010	30.2	12.6	2110	218	631.5	15,245	57.6	278
	29 November 2018	12.7	630	51	87	12,503	79.7	12.5	610	100	39.1	105,733	84.1	9

T—temperature; CO_2_—carbon dioxide; BDG—β-(1,3)-D-glucan.

**Table 2:** The influence of tourists on measured variables (median concentration of microorganisms, CO_2_ and BDG) in air samples from 2017 in Lepe jame (Postojnska jama), Vivarium (Postojnska jama) and Šotor (Škocjanske jame).

Location		CO_2_ [ppm]	*p*	Microbes (Culture) [CFU/m^3^]	*p*	BDG [pg/m^3^]	*p*
Lepe jame	Before tourists	990	0.400	103	0.463	18.25	0.068
	After tourists	1015		178.5		24.85	
Vivarium	Before tourists	955	0.344	152	0.345	24.45	0.138
	After tourists	1045		333		28.45	
Šotor	Before tourists	1055	0.102	140	0.046	49.90	0.249
	After tourists	1075		331		12.40	

CO_2_—carbon dioxide; BDG—β-(1,3)-D-glucan.

**Table 3:** Number and percentage of microbial isolates identified (MALDI score ≥ 1.70, genus level identification) per sampling site (Lepe jame, Vivarium and Šotor) and overall.

		Lepe Jame	Vivarium	Šotor	Locations Combined
Non-identified microorganisms (CFU/m^3^)	Before tourists	692	911	571	2174
	After tourists	566	1152	865	2583
All isolated microorganisms (CFU/m^3^)	Before tourists	1202	1571	1123	3896
	After tourists	1863	3792	2160	7815
% of identified microorganisms	Before tourists	42.4	42.0	49.2	44.2
	After tourists	69.6	69.6	60.0	66.9

**Table 4:** Potential microbial indicators of cave anthropisation (MALDI-TOF MS score value 2.00, species level identification).

Microorganisms	Sampling Sites *	Risk Group (Country)	Human Microbiota
**Bacteria**			
*Dermacoccus* spp.	2	-	skin, scalp
*Micrococcus luteus*	1, 2, 3	2 (BE, CH, DE, NIH)	skin
*Moraxella osloensis*	1	-	skin, mucosae
*Staphylococcus capitis*	1	-	skin, scalp
*Staphylococcus epidermidis*	1, 2, 3	2 (BE, CH, DE)	skin, nasopharynx
*Staphylococcus haemolyticus*	1, 2	2 (CH, DE)	skin
*Staphylococcus hominis*	3	-	skin
*Staphylococcus lugdunensis*	1, 2	2 (BE, CH, DE)	skin
*Staphylococcus saprophyticus*	2, 3	2 (BE, CA, CH, DE)	skin
*Staphylococcus warneri*	1, 2, 3	-	skin
*Streptococcus mitis*	1, 2	2 (BE, CH, DE, NIH)	oropharynx
**Fungi**			
*Naganishia diffluens*	1, 2	-	skin
*Naganishia liquefaciens*	2, 3	-	skin
*Rhodotorula mucilaginosa*	2, 3	1 (DE)	skin, oropharynx

BE—Belgium; CA—Canada; CH—Switzerland; DE—Germany; NIH—National Institutes of Health. * 1: Postojnska jama–Lepe jame, 2: Postojnska jama–Vivarium, 3: Škocjanske jame–Šotor.

## Data Availability

Data is contained within the article or supplementary material.

## Supplementary Materials

The following supporting information can be downloaded at: https://www.mdpi.com/article/10.3390/microorganisms11102381/s1, Table S1: Bacterial and fungal isolates (MALDI Score ≥ 2.00) from the Lepe jame sampling site in Postojnska jama; Table S2: Bacterial and fungal isolates (MALDI Score ≥ 2.00) from the Vivarium sampling site in Postojnska jama; Table S3: Bacterial and fungal isolates (MALDI Score ≥ 2.00) from the Šotor sampling site in Škocjanske jame; Table S4: Bacterial isolates (MALDI Score ≥ 2.00) from the sampling sites Lepe jame and the Vivarium in Postojnska jama and from the sampling site Šotor in Škocjanske jame, their risk group assignment and typical habitat; Table S5: Fungal isolates (MALDI Score ≥ 2.00) from the sampling sites Lepe jame and the Vivarium in Postojnska jama and from the sampling site Šotor in Škocjanske jame, their risk group assignment and typical habitat.
