# Supplementary material for: Effects of Anthropogenic Disturbance and Seasonal Variation on Aerobiota in Highly Visited Show Caves in Slovenia
Source: Microorganisms. 2023 Sep 23;11(10):2381. doi: 10.3390/microorganisms11102381 (PMC10608856; doi:10.3390/microorganisms11102381)
Supplement: Supplementary file 1 [file microorganisms-11-02381-s001.zip › Tables S1-S5.pdf]

Table S1: Bacterial and fungal isolates (MALDI Score ≥ 2.00) from the Lepe jame sampling site in Postojnska jama. Concentrations (CFU/m^3) and percentages (%) of isolates based on flow cytometry results.

		Lepe jame (CFU/m^3 (%))							
**Bacteria**		**February 2017**	**March 2017**	**May 2017**	**August 2017**	**October 2017**	**Decembre 2017**	**August 2018**	**Novembre 2018**
Aerococcus viridans	Before tourists	NP	NP	NP	NP	NP	NP	NP	NP
	After tourists	NP	NP	NP	NP	NP	NP	15 (0.19)	NP
Arthrobacter oxydans	Before tourists	NP	NP	NP	NP	NP	NP	NP	NP
	After tourists	NP	17 (0.04)	NP	NP	NP	NP	NP	NP
Arthrobacter tumbae	Before tourists	NP	17 (0.06)	NP	NP	NP	NP	NP	NP
	After tourists	NP	NP	NP	NP	NP	NP	NP	NP
Bacillus cereus	Before tourists	NP	17 (0.06)	NP	NP	NP	NP	NP	4 (0.05)
	After tourists	NP	NP	NP	NP	NP	17 (0.27)	NP	NP
Bacillus pumilus	Before tourists	NP	NP	NP	17 (0.04)	NP	NP	NP	NP
	After tourists	NP	17 (0.04)	NP	17 (0.04)	NP	NP	NP	NP
Bacillus simplex	Before tourists	NP	NP	NP	17 (0.04)	NP	NP	NP	NP
	After tourists	NP	NP	NP	NP	NP	17 (0.27)	NP	NP
Brevibacterium aurantiacum	Before tourists	NP	NP	NP	NP	NP	NP	NP	NP
	After tourists	NP	NP	NP	17 (0.04)	NP	NP	NP	NP
Brevibacterium sp.	Before tourists	NP	NP	NP	NP	NP	67 (0.73)	NP	NP
	After tourists	NP	17 (0.04)	NP	17 (0.04)	NP	NP	NP	NP
Brevundimonas intermedia	Before tourists	NP	NP	NP	NP	NP	17 (0.19)	NP	NP
	After tourists	NP	NP	NP	NP	NP	NP	NP	NP
Chryseobacterium sp.	Before tourists	NP	NP	NP	17 (0.04)	NP	NP	NP	NP
	After tourists	NP	NP	NP	NP	NP	NP	NP	NP
Jeotgalicoccus sp.	Before tourists	NP	NP	NP	NP	NP	NP	NP	NP
	After tourists	NP	NP	NP	17 (0.04)	NP	NP	NP	NP

Kocuria rhizophila	Before tourists	NP	17 (0.06)	NP	33 (0.8)	NP	NP	NP	NP
	After tourists	NP	NP	NP	NP	NP	17 (0.27)	NP	NP
Kytococcus sedentarius	Before tourists	NP	NP	NP	NP	NP	NP	NP	NP
	After tourists	NP	NP	NP	17 (0.04)	NP	NP	NP	NP
Microbacterium paraoxydans	Before tourists	NP	NP	NP	NP	NP	NP	NP	NP
	After tourists	NP	NP	NP	THI	NP	NP	NP	NP
Micrococcus luteus	Before tourists	NP	NP	17 (0.16)	134 (0.32)	NP	NP	NP	2 (0.02)
	After tourists	10 (0.15)	50 (0.13)	17 (0.13)	251 (0.6)	17 (0.21)	117 (1.84)	83 (1.04)	NP
Moraxella osloensis	Before tourists	NP	NP	NP	NP	NP	NP	NP	NP
	After tourists	17 (0.26)	NP	17 (0.13)	NP	NP	NP	8 (0.10)	NP
Moraxella sp.	Before tourists	NP	NP	NP	NP	NP	NP	NP	NP
	After tourists	NP	NP	NP	17 (0.04)	NP	NP	NP	NP
Oerskovia turbata	Before tourists	NP	NP	NP	NP	NP	NP	NP	NP
	After tourists	NP	NP	NP	NP	NP	NP	NP	33 (0.07)
Paenibacillu urinalis	Before tourists	NP	NP	NP	NP	NP	NP	NP	NP
	After tourists	NP	NP	NP	NP	17 (0.21)	NP	NP	NP
Paracoccus yeei	Before tourists	NP	NP	NP	NP	NP	NP	NP	NP
	After tourists	NP	17 (0.04)	NP	NP	17 (0.21)	NP	NP	NP
Pseudomonas sp.	Before tourists	NP	NP	NP	NP	NP	NP	NP	NP
	After tourists	NP	NP	NP	NP	NP	NP	8 (0.10)	NP
Rhodococcus erytropolis	Before tourists	NP	NP	NP	NP	NP	NP	NP	NP
	After tourists	NP	NP	NP	NP	NP	NP	NP	50 (0.11)
Roseomonas mucosa	Before tourists	NP	8 (0.03)	NP	NP	NP	NP	1 (0.01)	NP
	After tourists	NP	NP	NP	NP	NP	NP	NP	NP
Staphylococcus capitis	Before tourists	NP	NP	NP	NP	NP	NP	NP	NP
	After tourists	NP	NP	NP	33 (0.08)	NP	NP	NP	NP
Staphylococcus epidermidis	Before tourists	NP	NP	NP	NP	NP	NP	NP	17 (0.20)

	After tourists	50 (0.76)	NP	NP	117 (0.28)	NP	NP	NP	NP
Staphylococcus equorum	Before tourists	NP	NP	NP	NP	NP	NP	NP	NP
	After tourists	NP	17 (0.04)	NP	67 (0.16)	NP	NP	NP	NP
Staphylococcus haemolyticus	Before tourists	NP	NP	NP	NP	NP	NP	NP	NP
	After tourists	NP	NP	NP	NP	NP	6 (0.09)	NP	NP
Staphylococcus lugdunensis	Before tourists	NP	NP	NP	NP	NP	NP	NP	NP
	After tourists	NP	NP	NP	17 (0.04)	NP	NP	NP	NP
Staphylococcus sp.	Before tourists	NP	NP	NP	NP	NP	NP	NP	NP
	After tourists	NP	NP	NP	NP	NP	NP	8 (0.10)	NP
Staphylococcus warneri	Before tourists	NP	NP	NP	NP	NP	THI	NP	NP
	After tourists	NP	NP	NP	NP	NP	THI	NP	NP
Streptococcus mitis	Before tourists	NP	NP	NP	NP	NP	NP	NP	17 (0.20)
	After tourists	NP	NP	NP	NP	NP	NP	NP	NP
Streptomyces lavendulae	Before tourists	NP	NP	NP	NP	33 (0.14)	NP	NP	NP
	After tourists	NP	NP	NP	NP	NP	NP	NP	NP
Streptomyces sp.	Before tourists	NP	NP	NP	NP	NP	NP	8 (0.08)	NP
	After tourists	NP	NP	NP	NP	NP	NP	NP	NP
Viridibacillus sp.	Before tourists	NP	NP	NP	NP	NP	NP	NP	NP
	After tourists	NP	NP	NP	NP	NP	NP	17 (0.21)	NP
**Fungi**									
Aspergillus fumigatus	Before tourists	17 (0.26)	NP	NP	NP	NP	NP	NP	NP
	After tourists	NP	NP	NP	NP	NP	NP	NP	NP
Aureobasidium pullulans	Before tourists	NP	NP	NP	33 (0.08)	NP	NP	NP	NP
	After tourists	NP	NP	NP	NP	NP	NP	NP	NP
Naganishia diffluens	Before tourists	NP	NP	NP	NP	NP	NP	NP	NP
	After tourists	NP	NP	NP	NP	THI	NP	NP	NP
Paecilomyces variotii	Before tourists	NP	NP	NP	NP	NP	NP	NP	NP
	After tourists	17 (0.26)	NP	NP	NP	NP	NP	NP	NP

**Summary of microbial identification for all sampling campaigns**									
Non-identified microorganisms	Before tourists	34 (0.52)	34 (0.11)	50 (0.48)	149 (0.36)	80 (0.35)	337 (3.70)	8 (0.08)	NP
	After tourists	94 (1.42)	34 (0.08)	17 (0.13)	200 (0.48)	17 (0.21)	153 (2.41)	34 (0.43)	17 (0.04)
Sum (before tourists)	Before tourists	51	93	67	400	113	421	17	40
Sum (after tourists)	After tourists	188	169	51	787	68	327	173	100
% of identified species	Before tourists	33.3	63.4	25.4	62.8	29.2	20.0	52.9	100
	After tourists	50.0	79.9	66.7	74.6	75.0	53.2	80.3	83
Quotient of concentration between after vs. before tourists samples		3.7	1.8	0.8	2.0	0.6	0.8	10.2	2.5

THI – Thioglycollate broth; NP – not present

Tabele S2: Bacterial and fungal isolates (MALDI Score ≥ 2.00) from the Vivarium sampling site in Postojnska jama. Concentrations (CFU/m^3) and percentages (%) of isolates based on flow cytometry results.

		**Vivarium (CFU/m^3 (%))**						
**Bacteria**		March 2017	May 2017	August 2017	October 2017	Decembre 2017	August 2018	Novembre 2018
Acinetobacter lwoffii	Before tourists	NP	NP	NP	NP	NP	NP	NP
	After tourists	NP	33 (0.25)	33 (0.07)	NP	NP	NP	33 (0.22)
Aerococcus viridans	Before tourists	NP	17 (0.19)	33 (0.08)	NP	NP	NP	NP
	After tourists	NP	NP	NP	NP	NP	NP	NP
Arthrobacter sp.	Before tourists	NP	NP	NP	86 (1.61)	NP	NP	NP
	After tourists	NP	NP	NP	NP	NP	NP	NP
Arthrobacter polychromogenes	Before tourists	NP	NP	NP	NP	NP	NP	NP
	After tourists	NP	NP	50 (0.11)	NP	NP	NP	NP

Bacillus cereus	Before tourists	NP	NP	NP	NP	NP	NP	3 (0.03)
	After tourists	17 (0.02)	NP	NP	86 (0.94)	NP	NP	NP
Bacillus sp.	Before tourists	NP	NP	17 (0.04)	NP	NP	11 (0.18)	NP
	After tourists	NP	NP	NP	NP	NP	NP	NP
Bacillus licheniformis	Before tourists	NP	NP	NP	NP	NP	NP	NP
	After tourists	NP	33 (0.25)	NP	84 (0.92)	THI	NP	NP
Bacillus pumilus	Before tourists	NP	NP	NP	NP	NP	NP	NP
	After tourists	NP	NP	NP	84 (0.92)	NP	NP	NP
Bacillus simplex	Before tourists	NP	NP	17 (0.04)	NP	NP	NP	NP
	After tourists	17 (0.02)	NP	NP	NP	NP	NP	NP
Brevibacterium aurantiacum	Before tourists	NP	NP	NP	NP	NP	NP	NP
	After tourists	33 (0.05)	NP	NP	NP	NP	NP	NP
Brevundimonas intermedia	Before tourists	NP	NP	NP	NP	NP	NP	NP
	After tourists	NP	NP	NP	NP	17 (0.11)	NP	NP
Citricoccus nitrophenolicus	Before tourists	NP	NP	NP	NP	NP	NP	NP
	After tourists	NP	NP	NP	NP	NP	NP	1 (0.01)
Dermacoccus sp.	Before tourists	NP	NP	NP	NP	NP	NP	NP
	After tourists	NP	33 (0.25)	NP	NP	NP	NP	NP
Dietzia maris	Before tourists	2 (0.004)	NP	NP	NP	NP	NP	NP
	After tourists	NP	NP	NP	NP	NP	NP	NP
Dietzia sp.	Before tourists	NP	NP	NP	NP	NP	NP	NP
	After tourists	17 (0.02)	NP	NP	NP	NP	NP	NP
Glutamicibacter bergerei	Before tourists	NP	NP	NP	NP	NP	NP	NP
	After tourists	NP	NP	NP	NP	NP	NP	17 (0.11)
Kocuria rhizophila	Before tourists	NP	NP	NP	NP	NP	NP	NP
	After tourists	NP	17 (0.13)	NP	NP	NP	NP	NP
Kytococcus sedentarius	Before tourists	3 (0.006)	NP	NP	NP	NP	NP	NP

	After tourists	NP	NP	17 (0.04)	NP	NP	NP	NP
Micrococcus luteus	Before tourists	17 (0.03)	17 (0.19)	7 (0.02)	NP	33 (0.12)	NP	5 (0.05)
	After tourists	33 (0.05)	618 (4.60)	150 (0.32)	150 (1.64)	NP	17 (0.25)	117 (0.78)
Pantoea agglomerans	Before tourists	NP	NP	NP	NP	NP	NP	NP
	After tourists	NP	NP	THI	NP	NP	NP	NP
Paracoccus yeei	Before tourists	17 (0.03)	NP	NP	NP	33 (0.12)	NP	NP
	After tourists	NP	17 (0.13)	NP	NP	NP	NP	NP
Pseudomonas sp.	Before tourists	NP	NP	NP	NP	84 (0.29)	NP	NP
	After tourists	NP	NP	NP	NP	NP	NP	NP
Psychrobacillus sp.	Before tourists	NP	NP	NP	NP	NP	NP	NP
	After tourists	NP	NP	NP	33 (0.36)	NP	NP	NP
Psychrobacter sp.	Before tourists	NP	NP	NP	NP	NP	NP	NP
	After tourists	NP	NP	NP	NP	NP	NP	33 (0.22)
Rhodococcus fascians	Before tourists	NP	17 (0.19)	NP	NP	NP	NP	NP
	After tourists	NP	NP	NP	NP	NP	NP	NP
Rothia aeria	Before tourists	NP	NP	NP	NP	NP	NP	NP
	After tourists	NP	NP	NP	NP	NP	NP	33 (0.22)
Sporosarcina sp.	Before tourists	NP	NP	NP	NP	NP	NP	NP
	After tourists	NP	NP	NP	NP	NP	NP	17 (0.11)
Staphylococcus epidermidis	Before tourists	17 (0.03)	NP	NP	NP	NP	NP	NP
	After tourists	NP	NP	NP	NP	NP	NP	NP
Staphylococcus equorum	Before tourists	NP	NP	NP	NP	NP	NP	NP
	After tourists	NP	50 (0.37)	33 (0.07)	NP	NP	NP	33 (0.22)
Staphylococcus haemolyticus	Before tourists	NP	NP	NP	NP	NP	NP	NP
	After tourists	NP	17 (0.13)	NP	NP	NP	NP	NP

Staphylococcus lugdunensis	Before tourists	NP	NP	NP	NP	NP	THI	NP
	After tourists	33 (0.05)	NP	NP	NP	NP	NP	NP
Staphylococcus saprophyticus	Before tourists	NP	NP	NP	NP	NP	NP	NP
	After tourists	NP	17 (0.13)	NP	NP	NP	NP	NP
Staphylococcus warneri	Before tourists	NP	NP	NP	NP	5 (0.02)	NP	NP
	After tourists	NP	NP	NP	NP	8 (0.05)	NP	NP
Streptococcus pseudopneumoniae	Before tourists	NP	NP	NP	NP	NP	NP	NP
	After tourists	NP	NP	NP	NP	NP	NP	301 (1.99)
**Fungi**								
Aspergillus versicolor	Before tourists	NP	NP	NP	NP	NP	NP	217 (2.37)
	After tourists	NP	NP	NP	NP	NP	NP	267 (1.77)
Cutaneotrichosporon curvatum	Before tourists	NP	NP	NP	NP	NP	NP	NP
	After tourists	NP	NP	NP	NP	NP	7 (0.10)	NP
Debaryomyces hansenii	Before tourists	NP	NP	NP	NP	NP	NP	NP
	After tourists	33 (0.05)	NP	NP	NP	NP	NP	NP
Naganishia diffluens	Before tourists	NP	NP	NP	NP	NP	NP	NP
	After tourists	NP	NP	NP	THI	NP	NP	NP
Naganishia liquefaciens	Before tourists	2 (0.004)	NP	NP	NP	NP	NP	NP
	After tourists	NP	NP	NP	NP	NP	NP	NP
Rhodotorula mucilaginosa	Before tourists	NP	NP	NP	NP	NP	NP	NP
	After tourists	NP	NP	1 (0.002)	NP	NP	NP	NP
**Summary of microbial identification for all sampling campaigns**								
Non-identified microorganisms	Before tourists	85 (0.17)	101 (1.14)	NP	557 (4.52)	167 (0.58)	NP	1 (0.01)
	After tourists	143 (0.20)	452 (3.37)	49 (0.12)	349 (3.82)	34 (0.23)	57 (0.82)	68 (0.45)
Sum (before tourists)	Before tourists	143	152	74	643	322	11	226
Sum (after tourists)	After tourists	326	1287	333	786	59	81	920

% of identified species	Before tourists	40.6	33.6	100.0	13.4	48.1	100.0	99.6
	After tourists	56.1	64.9	85.3	55.6	42.4	29.6	92.6
Quotient of concentration between after vs. before tourists samples		2.3	8.5	4.5	1.2	0.2	7.4	4.1

THI – Thioglycollate broth; NP – not present

Tabele S3: Bacterial and fungal isolates (MALDI Score ≥ 2.00) from the Šotor sampling site in Škocjanske jame. Concentrations (CFU/m^3) and percentages (%) of isolates based on flow cytometry results.

Genus		Šotor (CFU/m^3 (%))							
		**February 2017**	**March 2017**	**May 2017**	**August 2017**	**October 2017**	**Decembre 2017**	**August 2018**	**Novembre 2018**
Acinetobacter guillouiae	Before tourists	NP	NP	NP	NP	7 (0.05)	NP	NP	NP
	After tourists	NP	NP	NP	NP	NP	NP	NP	NP
Acinetobacter sp.	Before tourists	NP	NP	NP	NP	NP	NP	NP	NP
	After tourists	NP	NP	17 (0.16)	17 (0.04)	NP	NP	NP	NP
Acinetobacter johnsonii	Before tourists	NP	NP	NP	17 (0.04)	NP	NP	NP	NP
	After tourists	NP	NP	NP	NP	NP	NP	NP	NP
Acinetobacter lwoffii	Before tourists	NP	NP	NP	NP	NP	NP	NP	NP
	After tourists	NP	NP	NP	6	17 (0.25)	NP	17 (0.11)	NP
Aerococcus viridans	Before tourists	NP	NP	NP	NP	NP	NP	THI	NP
	After tourists	NP	NP	NP	33 (0.07)	NP	NP	NP	NP
Arthrobacter oxydans	Before tourists	NP	NP	NP	NP	17 (0.11)	NP	NP	NP
	After tourists	NP	NP	NP	NP	NP	NP	NP	NP
Arthrobacter polychromogenes	Before tourists	NP	NP	NP	NP	NP	NP	8 (0.03)	NP
	After tourists	NP	NP	NP	17 (0.04)	NP	NP	NP	NP
Arthrobacter sulfonivorans	Before tourists	NP	NP	NP	NP	NP	NP	NP	NP
	After tourists	NP	NP	NP	33 (0.07)	NP	NP	NP	NP

Bacillus cereus	Before tourists	NP	NP	17 (0.16)	NP	NP	NP	NP	NP
	After tourists	NP	NP	17 (0.16)	NP	NP	NP	NP	NP
Bacillus licheniformis	Before tourists	NP	NP	NP	NP	NP	NP	NP	NP
	After tourists	117 (0.48)	NP	NP	NP	NP	NP	NP	NP
Bacillus sp.	Before tourists	NP	NP	NP	NP	NP	NP	THI	NP
	After tourists	NP	NP	NP	NP	NP	17 (0.24)	NP	NP
Bacillus muralis	Before tourists	NP	NP	NP	NP	NP	NP	NP	NP
	After tourists	NP	NP	NP	17 (0.04)	NP	NP	NP	NP
Bacillus novalis	Before tourists	NP	NP	NP	NP	NP	NP	NP	1 (0.008)
	After tourists	NP	NP	NP	NP	NP	NP	NP	NP
Bacillus simplex	Before tourists	NP	7 (0.03)	NP	NP	NP	NP	8 (0.03)	NP
	After tourists	NP	8 (0.03)	NP	17 (0.04)	NP	NP	NP	NP
Brevibacterium sp.	Before tourists	NP	NP	NP	NP	NP	NP	NP	NP
	After tourists	NP	NP	NP	NP	THI	NP	NP	NP
Dietzia maris	Before tourists	NP	NP	NP	NP	NP	NP	8 (0.03)	NP
	After tourists	NP	NP	NP	NP	NP	NP	NP	NP
Kocuria sp.	Before tourists	NP	NP	NP	33 (0.07)	NP	NP	NP	NP
	After tourists	NP	NP	NP	NP	NP	NP	NP	NP
Kocuria rhizophila	Before tourists	NP	NP	NP	NP	NP	NP	NP	NP
	After tourists	NP	NP	17 (0.16)	NP	NP	NP	33 (0.22)	NP
Massilia timonae	Before tourists	NP	NP	NP	NP	NP	NP	NP	NP
	After tourists	NP	NP	NP	17 (0.04)	NP	NP	NP	NP
Microbacterium sp.	Before tourists	NP	NP	NP	NP	17 (0.11)	NP	NP	NP
	After tourists	NP	NP	NP	NP	NP	NP	NP	NP
Micrococcus sp.	Before tourists	NP	NP	NP	NP	NP	NP	NP	NP
	After tourists	NP	17 (0.07)	NP	NP	NP	NP	NP	NP
Micrococcus luteus	Before tourists	50 (0.23)	NP	NP	67 (0.15)	NP	THI	45 (0.18)	33 (0.26)

	After tourists	33 (0.14)	18 (0.08)	84 (0.81)	67 (0.15)	84 (1.22)	NP	92 (0.60)	83 (0.08)
Pseudomonas antarctica	Before tourists	NP	NP	NP	NP	NP	NP	NP	NP
	After tourists	17 (0.07)	NP	NP	NP	NP	NP	NP	NP
Pseudomonas extremorientalis	Before tourists	NP	NP	NP	NP	NP	NP	NP	NP
	After tourists	33 (0.14)	NP	NP	NP	NP	NP	NP	NP
Pseudomonas fluorescens	Before tourists	THI	NP	NP	NP	NP	NP	NP	NP
	After tourists	NP	NP	NP	NP	NP	NP	NP	NP
Pseudomonas syriNPae	Before tourists	33 (0.15)	NP	NP	NP	NP	NP	NP	NP
	After tourists	NP	NP	NP	NP	NP	NP	NP	NP
Pseudomonas tolaasii	Before tourists	NP	NP	NP	NP	NP	NP	NP	NP
	After tourists	33 (0.14)	NP	NP	NP	NP	NP	NP	NP
Pseudomonas sp.	Before tourists	17 (0.08)	NP	NP	NP	NP	NP	NP	NP
	After tourists	NP	NP	NP	NP	NP	NP	NP	NP
Rahnella aquatilis	Before tourists	30 (0.14)	NP	NP	NP	NP	NP	NP	NP
	After tourists	NP	NP	NP	NP	NP	NP	NP	NP
Rhizobium rubi	Before tourists	NP	NP	NP	NP	NP	NP	NP	NP
	After tourists	17 (0.07)	NP	NP	NP	NP	NP	NP	NP
Roseomonas mucosa	Before tourists	NP	NP	NP	NP	NP	NP	NP	17 (0.14)
	After tourists	NP	NP	NP	NP	NP	NP	NP	17 (0.02)
Solibacillus sp.	Before tourists	NP	NP	NP	NP	NP	NP	NP	NP
	After tourists	NP	NP	NP	NP	4 (0.06)	NP	NP	NP
Sphingomonas sp.	Before tourists	NP	NP	NP	NP	NP	NP	8 (0.03)	NP
	After tourists	NP	NP	NP	NP	NP	NP	NP	NP
Staphylococcus epidermidis	Before tourists	NP	NP	17 (0.16)	NP	17 (0.11)	NP	NP	NP
	After tourists	NP	NP	33 (0.32)	NP	NP	NP	NP	NP
Staphylococcus hominis	Before tourists	NP	8 (0.03)	NP	33 (0.07)	NP	NP	NP	NP
	After tourists	NP	NP	NP	NP	NP	NP	NP	NP

Staphylococcus saprophyticus	Before tourists	NP	NP	NP	NP	NP	NP	NP	NP
	After tourists	NP	NP	17 (0.16)	NP	NP	NP	NP	NP
Staphylococcus warneri	Before tourists	NP	NP	NP	NP	NP	3 (0.06)	NP	NP
	After tourists	NP	NP	NP	NP	NP	4 (0.06)	NP	NP
Staphylococcus sp.	Before tourists	17 (0.08)		NP	NP	NP	NP	NP	NP
	After tourists	NP	NP	NP	NP	NP	NP	NP	NP
Stenotrophomonas sp.	Before tourists	NP	NP	NP	NP	NP	NP	NP	NP
	After tourists	50 (0.21)	NP	NP	NP	NP	NP	NP	NP
Streptomyces sp.	Before tourists	NP	NP	NP	NP	17 (0.11)	NP	NP	NP
	After tourists	NP	NP	NP	NP	NP	NP	NP	NP
**Fungi**									
Aureobasidium pullulans	Before tourists	NP	NP	NP	NP	NP	NP	NP	NP
	After tourists	NP	NP	NP	NP	NP	NP	17 (0.11)	NP
Cutaneotrichosporon curvatum	Before tourists	NP	NP	NP	NP	NP	NP	NP	NP
	After tourists	NP	NP	NP	17 (0.04)	33 (0.48)	NP	25 (0.16)	NP
Naganishia liquefaciens	Before tourists	NP	NP	NP	NP	NP	NP	NP	NP
	After tourists	NP	NP	NP	NP	50 (0.73)	NP	NP	NP
Rhodotorula mucilaginosa	Before tourists	NP	NP	NP	NP	NP	NP	NP	NP
	After tourists	NP	NP	NP	33 (0.07)	NP	NP	NP	NP
**Summary of microbial identification for all sampling campaigns**									
Non-identified microorganisms	Before tourists	68 (0.32)	50 (0.19)	17 (0.16)	101 (0.22)	318 (2.15)	17 (0.35)	NP	NP
	After tourists	27 (0.11)	17 (0.07)	150 (1.45)	185 (0.41)	351 (5.10)	101 (1.41)	34 (0.22)	NP
Sum (before tourists)	Before tourists	215	65	51	251	393	20	77	51
Sum (after tourists)	After tourists	327	60	335	459	539	122	218	100
% of identified species	Before tourists	68,4	23,1	66,7	59,8	19,1	15,0	100,0	100,0
	After tourists	91,7	71,7	55,2	59,7	34,9	17,2	84,4	100,0

Quotient of concentration between after vs. before tourists samples	1,5	0,9	6,6	1,8	1,4	6,1	2,8	2,0

THI – Thioglycollate broth; NP – not present

Tabele S4: Bacterial isolates (MALDI Score ≥ 2.00) from the sampling sites Lepe jame and Vivarium in Postojnska jama and from the sampling site Šotor in Škocjanske jame, their risk group assignment and typical habitat. Concentrations (CFU/m³) and percentages (%) of isolates based on flow cytometry results.

**Bacterial species**	**Lepe jame**		**Vivarium**		**Šotor**		**Locations Combined**		**Risk Group**	**Typical Habitat**
	Bacteria (CFU/m³ (%))		Bacteria (CFU/m³ (%))		Bacteria (CFU/m³ (%))		Bacteria (CFU/m³)			
	Before tourists	After tourists	Before tourists	After tourists	Before tourists	After tourists	Before tourists	After tourists		
Acinetobacter guillouiae	NP	NP	NP	NP	7 (0.62)	NP	0-7	NP	-	ubiquitous, gasworks, humans (skin)
Acinetobacter johnsonii	NP	NP	NP	NP	17	NP	0-17	NP	2 (AU, CH, DE)	humans (skin), animals, activated sludge, food
Acinetobacter lwoffii	NP	NP	NP	99 (2.61)	NP	40 (1.85)	NP	0-99	2 (AU, CH, DE)	ubiquitous, food, humans (skin)
Aerococcus viridans	NP	15 (0.81)	50 (3.18)	NP	NP	33 (1.53)	0-50	0-33	2 (BE, CH, DE)	Humans (urinary tract), animals (lobsters), seawater
Arthrobacter oxydans	NP	17 (0.91)	NP	NP	17 (1.51)	NP	0-17	0-17	-	ubiquitous, soil, plants
Arthrobacter polychromogenes	NP	NP	NP	50 (1.32)	8 (0.71)	17 (0.79)	0-8	0-50	-	ubiquitous, soil, plants

Arthrobacter sulfonivorans	NP	NP	NP	NP	NP	33 (1.53)	NP	0-33	-	ubiquitous, soil, plants
Arthrobacter tumbae	17 (1.41)	NP	NP	NP	NP	NP	0-17	NP	-	ubiquitous, soil, plants
Bacillus cereus	21 (1.75)	17 (0.91)	3 (0.19)	103 (2.72)	17 (1.51)	17 (0.79)	3-21	17-103	2 (AU, CH, DE, UK)	humans, soil, food
Bacillus licheniformis	NP	NP	NP	117 (3.09)	NP	117 (5.42)	NP	0-117	-	soil, food, animals (birds)
Bacillus megaterium	NP	NP	NP	NP	THI	NP	NP	NP	-	soil, seawater, food, animals (insects)
Bacillus novalis	NP	NP	NP	NP	1 (0.09)	NP	0-1	NP	-	soil, hay fields
Bacillus pumilus	17 (1.41)	34 (1.83)	NP	84 (2.22)	NP	NP	0-17	0-84	-	soil, animals, food (milk)
Bacillus simplex	17 (1.41)	17 (0.91)	17 (1.08)	17 (0.45)	15 (1.34)	25 (1.16)	15-17	17-25	-	soil, plants
Brevibacterium aurantiacum	NP	17 (0.91)	NP	33 (0.87)	NP	NP	NP	0-33	-	food (milk and cheese)
Brevundimonas intermedia	17 (1.41)	NP	NP	17 (0.45)	NP	NP	0-17	0-17	-	ubiquitous, seawater
Citricoccus nitrophenolicus	NP	NP	NP	1 (0.03)	NP	NP	NP	0-1	-	wastewater
Dietzia maris	NP	NP	2 (0.13)	NP	8 (0.71)	NP	0-8	NP	-	humans, animals, seawater
Glutamicibacter bergerei	NP	NP	NP	17 (0.45)	NP	NP	NP	0-17	-	soil
Kocuria rhizophila	50 (4.16)	17 (0.91)	NP	17 (0.45)	NP	50 (2.31)	0-50	17-50	-	Soil, sand, human and animal skin
Kytococcus sedentarius	NP	17 (0.91)	3 (0.19)	17 (0.45)	NP	NP	0-3	0-17	-	aquatic, humans (skin)
Massilia timonae	NP	NP	NP	NP	NP	17 (0.79)	NP	0-17	-	Plants (maize), humans
Microbacterium paraoxydans	NP	THI	NP	NP	NP	NP	NP	NP	-	Humans, plastic

Micrococcus luteus	153 (12.7)	545 (29.3)	79 (5.02)	1085 (28.61)	195 (17.36)	461 (21.34)	79-195	545-1085	2 (BE, CH, DE, NIH)	soil, aquatic, air, human and animal skin
Moraxella osloensis	NP	42 (2.25)	NP	NP	NP	NP	NP	0-42	-	Human skin and mucosae
Oerskovia turbata	NP	33 (1.77)	NP	NP	NP	NP	NP	0-33	-	Soil, human
Paenibacillus urinalis	NP	17 (0.91)	NP	NP	NP	NP	NP	0-17	-	Human (urin)
Pantoea agglomerans	NP	NP	NP	THI	NP	NP	NP	NP	2 (CH)	Plants, animals (insects), dust
Paracoccus yeei	NP	34 (1.83)	50 (3.18)	17 (0.45)	NP	NP	0-50	0-34	2 (AU)	Soil, brines
Pseudomonas antarctica	NP	NP	NP	NP	NP	17 (0.79)	NP	0-17	2 (AU)	Aquatic, soil, ice, cyanobacterial mats
Pseudomonas extremorientalis	NP	NP	NP	NP	NP	33 (1.53)	NP	0-33	2 (AU)	Aquatic, soil, plants
Pseudomonas fluorescens	NP	NP	NP	NP	THI	NP	NP	NP	2 (AU)	Soil, plants, humans, animals
Pseudomonas syringae	NP	NP	NP	NP	33 (2.94)	NP	0-33	NP	2 (AU)	Plants
Pseudomonas tolaasii	NP	NP	NP	NP	NP	33 (1.53)	NP	0-33	2 (AU)	Mushrooms
Rahnella aquatilis	NP	NP	NP	NP	30 (2.67)	NP	0-30	NP	-	Aquatic, plants
Rhizobium rubi	NP	NP	NP	NP	NP	17 (0.79)	NP	0-17	-	Plants, soil
Rhodococcus erythropolis	NP	50 (2.68)	NP	NP	NP	NP	NP	0-50	-	Soil, seawater, sediments
Rhodococcus fascians	NP	NP	17 (1.08)	NP	NP	NP	0-17	NP	-	Plants
Roseomonas mucosa	9 (0.75)	NP	NP	NP	17 (1.51)	17 (0.79)	0-17	0-17	-	Humans (skin)
Rothia aeria	NP	NP	NP	33 (0.87)	NP	NP	NP	0-33	-	Air, humans (oral mucosa)
Staphylococcus capitis	NP	33 (1.77)	3 (0.19)	NP	NP	NP	0-3	0-33	-	Animals, humans (skin, scalp)

Staphylococcus epidermidis	17 (1.41)	167 (8.96)	17 (1.08)	NP	34 (3.03)	33 (1.53)	17-34	0-167	2 (BE, CH, DE)	Animals, humans (skin, nasopharynx), fermented sausages
Staphylococcus equorum	NP	84 (4.51)	NP	116 (3.06)	NP	NP	NP	0-116	-	Fermented food, animals (horses, cattle)
Staphylococcus haemolyticus	NP	6 (0.32)	NP	17 (0.45)	NP	NP	NP	0-17	2 (CH, DE)	Fermented food, milk, animals (horses, cattle, cats), humans (skin)
Staphylococcus hominis	NP	NP	NP	NP	41 (3.65)	NP	0-41	NP	-	Fermented food, goat milk, animals (cats, dogs, goats), humans (skin)
Staphylococcus lugdunensis	NP	17 (0.91)	NP	33 (0.87)	NP	NP	NP	0-33	2 (BE, CH, DE)	Animals (cats, dogs), humans (skin)
Staphylococcus saprophyticus	NP	NP	NP	17 (0.45)	NP	17 (0.79)	NP	0-17	2 (BE, CA, CH, DE)	Animals (horses, cattle), humans (skin)
Staphylococcus warneri	THI	THI	5 (0.32)	8 (0.21)	3 (0.27)	4 (0.19)	0-5	4-5	-	Fermented food, animals (cats, dogs, horses), humans (skin)
Streptococcus mitis	17 (1.41)	NP	NP	NP	NP	NP	0-17	NP	2 (BE, CH, DE, NIH)	Humans (oropharynx)
Streptococcus pseudopneumoniae	NP	NP	NP	301 (7.94)	NP	NP	NP	0-301	-	Humans (oropharynx)
Streptomyces lavendulae	33 (2.75)	NP	NP	NP	NP	NP	0-33	NP	-	Soil

BE—Belgium; CA—Canada; CH—Switzerland; DE—Germany; NIH – National Institutes of Health; THI – Thioglycollate broth; NP – not present

Tabela S5: Fungal isolates (MALDI Score ≥ 2.00) from the sampling sites Lepe jame and Vivarium in Postojnska jama and from the sampling site Šotor in Škocjanske jame, their risk group assignment and typical habitat. Concnetrations (CFU/m³) and percentages (%) of isolates based on flow cytometry results.

**Fungal isolates**	**Lepe jame**		**Vivarium**		**Šotor**		**Locations Combined**		**Risk Group**	**Typical habitat**
	Fungi (CFU/m³ (%))		Fungi (CFU/m³ (%))		Fungi (CFU/m³ (%))		Fungi (CFU/m³)			
	Before tourists	After tourists	Before tourists	After tourists	Before tourists	After tourists	Before tourists	After tourists		
*Aspergillus fumigatus**	17 (1.41)	NP	NP	NP	NP	NP	0-17	NP	2 (AU, CA, CH, DE, EU)	Ubiquitous, air, dust, plants
*Aspergillus versicolor**	NP	NP	217 (13.79)	267 (7.04)	NP	NP	0-217	0-267	2 (UK)	Ubiquitous, air, dust, plants
Aureobasidium pullulans	33 (2.75)	NP	NP	NP	NP	17 (0.79)	0-33	0-17	-	Ubiquitous, plants
Cutaneotrichosporon curvatum	NP	NP	NP	7 (0.18)	NP	75 (3.47)	NP	0-82	-	Air, aquatic, wood, food
Debaryomyces hansenii	NP	NP	NP	33 (0.87)	NP	NP	NP	0-33	-	Seawater, fermented food
Naganishia diffluens	NP	THI	NP	THI	NP	NP	NP	NP	-	Air, aquatic, wood, food, humans (skin)
Naganishia liquefaciens	NP	NP	2 (0.13)	NP	NP	50 (2.31)	0-2	0-50	-	Air, aquatic, wood, food, humans (skin)
*Paecilomyces variotii**	NP	17 (0.91)	NP	NP	NP	NP	NP	0-17	2 (BE, CH, DE)	Air, wood, soil, food
Rhodotorula mucilaginosa	NP	NP	NP	1 (0.03)	NP	33 (1.53)	NP	0-34	1 (DE)	Air, soil, food (cheese, milk), humans (skin, oropharynx)
All Fungi	50 (4.16)	17 (0.91)	219 (13.91)	308 (8.13)	NP	175 (8.10)	0-217	0-267	-	-

BE—Belgium; CA—Canada; CH—Switzerland; DE—Germany; EU – European Union; NIH – National Institutes of Health; THI – Thioglycollate broth; NP – not present
* Identification is based on the morphological characteristics of the isolates.
